# Comparing the validity of different ICD coding abstraction strategies for sepsis case identification in German claims data

**DOI:** 10.1371/journal.pone.0198847

**Published:** 2018-07-30

**Authors:** Carolin Fleischmann-Struzek, Daniel O. Thomas-Rüddel, Anna Schettler, Daniel Schwarzkopf, Angelika Stacke, Christopher W. Seymour, Christoph Haas, Ulf Dennler, Konrad Reinhart

**Affiliations:** 1 Integrated Research and Treatment Center, Center for Sepsis Control and Care (CSCC), Jena University Hospital, Jena, Germany; 2 Department for Anesthesiology and Intensive Care Medicine, Jena University Hospital, Jena, Germany; 3 Department of Critical Care Medicine, University of Pittsburgh School of Medicine, Pittsburgh, Pennsylvania, United States of America; 4 Clinical Research, Investigation, and Systems Modeling of Acute illness (CRISMA) Center, Pittsburgh, Pennsylvania, United States of America; 5 Division of Medical Controlling, Jena University Hospital, Jena, Germany; University Medical Center Goettingen, GERMANY

## Abstract

**Introduction:**

Administrative data are used to generate estimates of sepsis epidemiology and can serve as source for quality indicators. Aim was to compare estimates on sepsis incidence and mortality based on different ICD-code abstraction strategies and to assess their validity for sepsis case identification based on a patient sample not pre-selected for presence of sepsis codes.

**Materials and methods:**

We used the national DRG-statistics for assessment of population-level sepsis incidence and mortality. Cases were identified by three previously published *International Statistical Classification of Diseases* (ICD) coding strategies for sepsis based on primary and secondary discharge diagnoses (clinical sepsis codes (R-codes), explicit coding (all sepsis codes) and implicit coding (combined infection and organ dysfunction codes)). For the validation study, a stratified sample of 1120 adult patients admitted to a German academic medical center between 2007–2013 was selected. Administrative diagnoses were compared to a gold standard of clinical sepsis diagnoses based on manual chart review.

**Results:**

In the validation study, 151/937 patients had sepsis. Explicit coding strategies performed better regarding sensitivity compared to R-codes, but had lower PPV. The implicit approach was the most sensitive for severe sepsis; however, it yielded a considerable number of false positives. R-codes and explicit strategies underestimate sepsis incidence by up to 3.5-fold. Between 2007–2013, national sepsis incidence ranged between 231-1006/100,000 person-years depending on the coding strategy.

**Conclusions:**

In the sample of a large tertiary care hospital, ICD-coding strategies for sepsis differ in their accuracy. Estimates using R-codes are likely to underestimate the true sepsis incidence, whereas implicit coding overestimates sepsis cases. Further multi-center evaluation is needed to gain better understanding on the validity of sepsis coding in Germany.

## Introduction

Acknowledging that sepsis is the leading cause of death from infection and affecting more than 30 million patients globally [1], the World Health Organization declared the prevention, diagnosis and management of sepsis as leading priority in its member states [2, 3]. For most countries, population-level sepsis incidence and mortality rates remain unknown, thus this resolution urges to implement measures of specific epidemiologic surveillance and “to apply and improve the use of the International Classification of Diseases system to establish the prevalence and profile of sepsis” [2]. In the US and several European countries, estimates on sepsis incidence are commonly drawn from retrospective studies based on hospital claims data using different International Classification of Diseases (ICD) codes for case identification [4, 5]. Administrative data are also increasingly used to compare risk-adjusted mortality rates in different conditions between health care providers [6–8]. In Germany, both the Initiative for Quality Medicine [9] and the German Quality Network Sepsis [10] provide their participating hospitals quality indicators on hospital mortality based on diagnostics-related-groups (DRG) data. Various ICD combinations emerged which attempt to capture sepsis patients. While explicit coding approaches use ICD codes for septicemia/sepsis [11], implicit coding approaches link infection and organ dysfunction codes to mirror clinical sepsis criteria [5]. Depending on the underlying codes, estimates on sepsis incidence and mortality differ considerably [12]. Efforts were made to validate ICD case identification strategies compared to a gold standard of manual patient chart review [13]. These studies have shown good specificity, but poor sensitivity of sepsis coding in claims data [14]. Recent population based studies from Scandinavia suggest that ICD abstraction may result in an up to 6-fold underestimation of the incidence of traditional severe sepsis and also in comparison to the newly proposed sepsis definition designated as “sepsis-3” [15] compared to medical record review [16, 17]. Existing validation studies mostly rely on small sample sizes, selective populations and restrict their review to charts selected based on the presence or absence of relevant ICD sepsis codes [13], an important source of bias, since sensitivity could not reliably be estimated by this approach. This is why we aimed compare validity of different ICD 10 code abstraction regarding a gold standard of manual patient chart review not pre-selected for presence of sepsis codes. Furthermore, no data exists in Germany on the variations in incidence and mortality due to different identification of sepsis cases in administrative data. Above that, it is yet unknown how patients identified using new “sepsis-3” definitions will be coded in administrative claims, perhaps impacting estimates of sepsis epidemiology. Further aims of this study were therefore to compare ICD code abstraction strategies’ estimates on sepsis incidence and mortality by using German hospital claims data, and to assess the concordance of “sepsis-3” definitions with cases identified by retrospective chart review.

## Materials and methods

### ICD coding strategies for sepsis

ICD coding strategies for sepsis in claims data were selected based on a review of international studies applying ICD abstraction strategies (S1 File). Codes for five main coding strategies were selected and translated from ICD-9 to ICD-10-German Modification (GM): for sepsis: I) R-codes, II) explicit approach (all sepsis codes = microbiological sepsis codes and R-codes); for severe sepsis: III) R-codes, (IV) explicit and organ dysfunction codes and V) implicit approach (presence of infection and organ dysfunction codes, Angus method [5]). R-codes for sepsis (R65.0!), severe sepsis (R65.1!) and septic shock (R57.2) are defined according to modified ACCP/SCCM consensus criteria [18]. In Germany, R65.0 and R65.1 were introduced in 2005; R57.2 in 2010. The use of R65.0 is restricted since 2007 a) to cases with positive blood culture and 2/4 positive SIRS criteria or b) in case of negative blood cultures only when 4/4 SIRS criteria are present. For specific codes, see S1 File.

### Validation of coding strategies

Validation of ICD coding strategies was performed against a gold standard of manual chart review of patients admitted to a large university hospital in Germany (Jena University Hospital). We planned the sample size for the validation sample based on considerations for the width of a two-sided 95% confidence interval (CI) for sensitivity of explicit sepsis codes. We expected a sensitivity of 10% [14] and aimed at a 5% margin of the CI. This resulted in a sample size estimation of n = 139 true cases with sepsis. Given an expected inpatient incidence of sepsis of 2.3% [5], we decided for a stratified sampling of patient charts. Cases were sampled from a 2 x 2 classification based on any need for ICU care after admission, and length of stay > or ≤ 6 days. The same number of patients was sampled from each stratum, thus resulting in higher sampling probabilities for patients with longer stay and ICU treatment. Based on considerations of the available resources to conduct the chart review, we sampled a total of 1120 charts from all adult hospitalizations admitted between 2007 and 2013. From the sample, every chart was reviewed for the presence of infection and sepsis independently by two investigators blinded to the patients’ administrative data using a structured protocol (S1 File), discrepancies were resolved by discussion and third review. Sepsis was defined according to modified ACCP/SCCM consensus criteria (“sepsis-1”) [19] based on all available patient data. Organ dysfunctions were evaluated for diagnosis of severe sepsis and were considered if most likely caused by sepsis. For each patient with infection, additional information on microbiology, ICU treatment, severity scores and further clinical data were extracted. In a separate review conducted in the same way, cases fulfilling the “sepsis-3” definitions were identified [15]. In case of missing clinical information, no Sequential [Sepsis-related] Organ Failure Assessment (SOFA) score points were assigned in the respective category. Furthermore, we identified patients with false negative or false positive ICD coding and evaluated reasons for incorrect coding based of in available information in the patient chart. The study was approved by the Jena University Hospital IRB (file 4333-02/15) and the Jena University Hospital data protection officer. The need for informed consent was waived, since only routinely obtained clinical data were used.

### German national incidence and outcomes of different coding abstraction strategies

National sepsis incidence and mortality were analyzed between 2007 and 2013 using the nationwide German DRG statistics, which includes inpatient claims data from all acute-care hospitals in Germany and is accessible by the Federal Statistical Office (S1 File). We identified septic patients (≥18y) by strategies I)-V) applied to the primary and all secondary discharge diagnoses, since there is no chronological or hierarchical order of secondary diagnoses in Germany. Comorbidities were defined using the Charlson Comorbiditiy Index [20]. Organ dysfunction codes were classified as cardiovascular, respiratory, central nervous system, renal, metabolic, hematologic and hepatic [5]. We used procedure codes to identify patients with ICU treatment, ventilation and hemodialysis (S1 File).

### Analyses

Interrater-agreement in the manual chart review was assessed by the S statistic for dichotomous data for the diagnosis of infection and by the S statistic with linear weighting for the diagnosis of sepsis (no sepsis, sepsis without organ dysfunction, severe sepsis/shock) [21], an improved alternative statistic to Fleiss kappa [22]. Characteristics of patients with and without sepsis were compared by standard descriptive statistics. Sensitivity, specificity, positive predictive value (PPV) and negative predictive value (NPV) of the coding strategies regarding the gold standard were calculated correcting for sampling weights. Confidence intervals on the 95% level (95% CI) were obtained by 2000 weighted bootstrap samples using the non-parametric percentile method. Cross-tabulation of cases identified by different strategies and the gold standard was done and depicted graphically using the UpSetR web application [23]. Patients with sepsis identified in national German DRG data were characterized by standard descriptive statistics. Incidence and mortality were compared between the years 2007–2013. The annual population-based incidence and mortality rate of sepsis was directly age- and sex-standardized to the German population structure as of 31st December 2010 based on nationwide population data provided by the Federal Statistical Office. Analyses were performed using IBM SPSS Statistics^®^ 20.0 and 23 and the statistical package R [24].

## Results

### Validation of ICD code abstraction strategies

**Fig 1:**
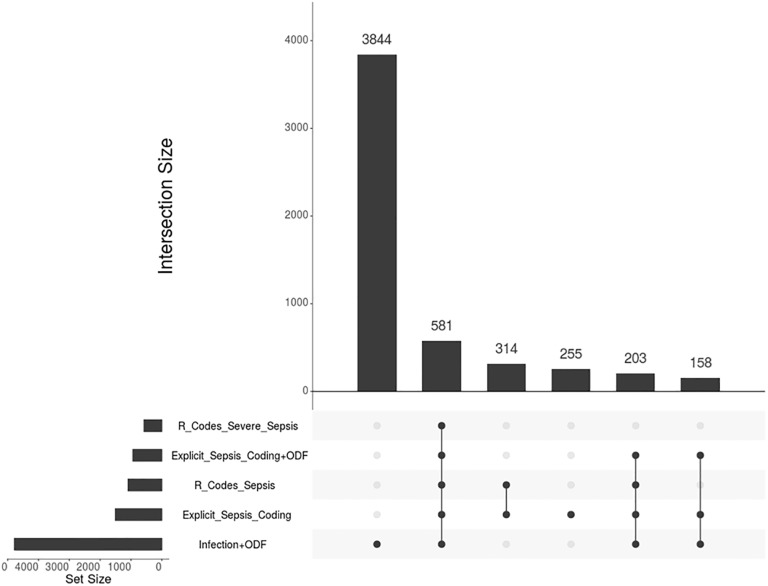
Intersecting sets of sepsis patients ≥18y in Germany 2007–2013 by different ICD code abstraction strategies; horizontal bars represent the numbers of patients identified by a specific coding strategy; vertical bars represent the number of patients identified by only one or several coding strategies indicated by dots with connecting lines (all numbers divided by 1000, generated with UpSetR[23]).

Of 1120 cases selected by stratified sampling 937 charts were available; 183 charts were inaccessible for accompanying parents, diagnostic or therapeutic day cases without inpatient charts or when charts were not available at the archive at the time of review. Inter-rater-agreements were high both for diagnosis of infection and sepsis (S [95% CI] of 0.87 [0.84, 0.90] and 0.93 [0.91, 0.95], respectively). 151 patients had sepsis, including 81 patients with severe sepsis/septic shock. For patient demographics, see Table 1. Among the strategies for sepsis and severe sepsis case identification, the explicit coding strategy yielded a substantially higher sensitivity value compared to R-codes, while having no decreased PPV (Table 2). The implicit coding strategy for severe sepsis showed the highest sensitivity, but yielded a substantial number of false positives, resulting in a positive predictive value of only 22.1%. After correction for sampling weights, we found an underestimation of cases by up to 3.5-fold by R codes and explicit coding. Implicit coding led to a 2.7-fold overestimation of severe sepsis cases. The overlap between cases identified by manual patient chart review and from administrative data is shown in Fig 1 and Figure B in S2 File, as well as an overview on reasons for false positive or negative coding.

**Table 1:** Demographics of patients identified by clinical chart review in a sample stratified by length of stay and ICU treatment (n = 937).

Characteristic	No infection	Infection w/o sepsis	Sepsis without organ dysfunction	Sepsis with organ dysfunction (severe sepsis)	Septic shock
cases	650	135	70	17	64
age (y, median [IQR])	61 [44–72]	70 [55–79]	70 [54–70]	68 [53–79]	70 [60–78]
male sex (%)	52	50	63	65	63
hospital mortality (%)	8.0	11.1	11.4	29.4	60.9
microbiological prove of infection (%)	n/a	62	62	71	83
blood culture rate (%)	0.2	30	46	76	94
positive blood cultures (%)	0	27	34	62	40
organ dysfunctions (number, median [IQR])	n/a	n/a	n/a	3 [3–4]	3 [2–4]
• encephalopathy (%)				43	50
• thrombocytopenia (%)				24	44
• respiratory failure (%)				65	52
• renal failure (%)				24	57
• metabolic acidosis (%)				24	66
organ replacement therapy according to OPS codes:					
• mechanical ventilation (%)	19	33	51	56	89
• renal replacement (%)	5	12	6	18	44
focus of infection					
• respiratory (%)	n/a	31	49	71	69
• abdominal (%)	n/a	19	21	12	39
• genitourinary (%)	n/a	30	16	12	27
• wound/soft tissue (%)	n/a	21	10	24	6
• pregnancy-related (%)	n/a	0	0	0	0
• device-related (%)	n/a	5	6	0	13
• other (%)	n/a	7	14	12	14
• unknown (%)	n/a	6	10	0	3

IQR = interquartile range

**Table 2:** Sensitivity, specificity, PPV and NPV of ICD abstraction strategies for sepsis and severe sepsis against a gold standard of manual patient chart review.

Characteristic	ICD abstraction strategies for sepsis (incl. severe sepsis)		ICD abstraction strategies for severe sepsis (incl. septic shock)		
	R codes (R65.0!, R65.1!, R57.2)	explicit sepsis coding*	R codes (R65.1!, R57.2)	explicit sepsis coding +organ dysfunction	implicit sepsis coding (infection + organ dys.)
Sensitivity (95% CI)	22.1% (15.2–29.1)	25.7% (19.2–33.1)	25.1% (16.0–34.6)	41.9% (30.9–51.9)	59.0% (48.1–69.1)
Specificity (95% CI)	99.6% (99.1–100)	99.6% (99.1–100)	99.6% (99.1–100)	99.4% (99.8–99.9)	95.7% (94.4–97.1)
PPV	77.2%	78.5%	56.1%	59.6%	22.1%
NPV	95.8%	95.9%	98.5%	98.8%	99.1%

* explicit sepsis codes: A02.1, A20.0, A20.7, A21.7, A22.7, A24.1, A26.7, A28.2, A32.7, A39.2, A39.3, A39.4, A39.1, A40.-, A41.-, A42.7, A48.3, B00.7, A54.8, B37.7, B37.6, B49, A49.9, R65.0!, R65.1!, R57.2

### Proposed new sepsis definitions

From 70 cases clinically defined as sepsis without organ dysfunction according to the “sepsis-1” criteria in the validation cohort, the majority (98%) had no organ dysfunction as defined by SOFA change and thus was classified non-septic according to the new definitions (Table H in S2 File). All severe sepsis patients (n = 17) met the “sepsis-3”-criteria. This was also true for septic shock patients (n = 64). However, n = 4 (8%) had no elevated lactate and thus did not meet the “sepsis-3”-criteria for septic shock.

### National estimates on sepsis incidence rates and mortality

**Fig 2:**
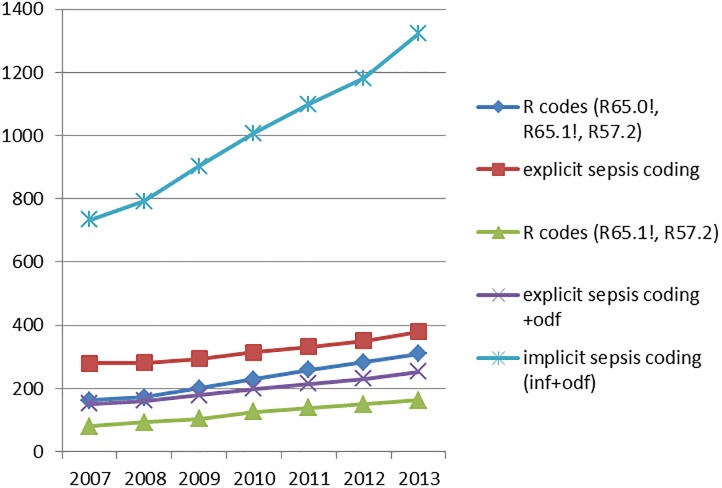
Course of hospital treated sepsis incidence ≥18y in Germany per 100,000 persons ≥18y per year by ICD code abstraction strategies, adjusted to the German population ≥18y in 2010.

Between 2007 and 2013 there was a steady increase in hospital treated sepsis incidence for any ICD coding abstraction strategy. The annual increase was most pronounced for implicit sepsis coding (Fig 2). The average annual sepsis incidence in adults ranged from 231/100,000 persons using R-codes, 318/100,000 using all sepsis codes (explicit coding strategy) to 1006/100,000 using any combination of infection and organ dysfunction (Table 3, Table D in S2 File). There was an increase in the absolute number of annual hospital deaths (Table B in S2 File) for all abstraction strategies despite decreasing case fatality rates (Table C and Figure A in S2 File). The average number of hospital deaths with sepsis per year ranged from 71 to 200/100,000 population depending on abstraction strategies (Table 3, Table E in S2 File). Sepsis cases identified using different abstraction strategies had differences in demographics, comorbidities, length of stay and mortality (Table 3). Of cases identified by any of these strategies, 72% of all cases met only the implicit case definition and had neither R nor explicit sepsis codes.

**Table 3:** Cases, incidence, mortality and demographics of sepsis patients ≥18y in Germany 2007–2013 by different ICD code abstraction strategies.

Characteristic	ICD abstraction strategies for sepsis (incl. severe sepsis)		ICD abstraction strategies for severe sepsis*		
	R codes (R65.0!, R65.1!, R57.2)	explicit sepsis coding*	R codes (R65.1!, R57.2)	explicit sepsis coding +odf**	implicit sepsis coding (inf+odf**)
cases 2007–2013	1,098,222	1,511,510	580,778	941,957	4,785,511
incidence / 100,000 population ≥18y / year	231	318	122	198	1006
hospital deaths / 100,000 population ≥18y / year	71	87	57	77	200
age (y, median [IQR])	72 [62–80]	72 [61–80]	72 [62–80]	72 [62–80]	75 [65–82]
male sex (%)	56	55	58	57	52
mortality (%)	30.8	27.5	47.0	38.7	19.8
ICU treatment (%)	37.4	31.8	53	45.2	25.0
CCI (median [IQR]	3 [1–4]	2 [1–4]	3 [1–5]	3 [1–5]	3 [1–4]
hospital LOS (d, median [IQR])	15 [8–28]	14 [8–27]	17 [8–33]	17 [8–32]	13 [7–22]

* explicit sepsis codes: A02.1, A20.0, A20.7, A21.7, A22.7, A24.1, A26.7, A28.2, A32.7, A39.2, A39.3, A39.4, A39.1, A40.-, A41.-, A42.7, A48.3, B00.7, A54.8, B37.7, B37.6, B49, A49.9, R65.0!, R65.1!, R57.2

** organ dysfunction

CCI = Charlson Comorbidity Index, IQR = interquartile range

## Discussion

Using manual patient chart review as gold standard in a single center validation study in a large tertiary care hospital in Germany, we found that current ICD abstraction strategies differ substantially in their accuracy of sepsis case identification in administrative data. There is a trade-off between sensitivity and positive predictive value across different strategies. Explicit coding strategies have a better positive prediction compared to implicit coding strategies, but show a limited sensitivity and may miss a relevant number of sepsis cases in administrative data. R-codes and explicit sepsis coding strategies may underestimate sepsis incidence by 3.5-fold and 3-fold, respectively. Severe sepsis incidence rates may also be underestimated by 2.2-fold and 1.4-fold when using R-codes or explicit strategy, whereas implicit strategies risk overestimation by 2.7-fold. Our findings are in accordance with the results of studies from Denmark, Sweden and the US, which found an underestimation of severe sepsis cases in administrative data [4, 16, 17]. In a recent population based study from Sweden [17], a minority of 15.6% of patients with clinically diagnosed sepsis according to “sepsis-3” was coded as sepsis. In the US, only 30.5% of sepsis cases identified by “sepsis-3” criteria in eletronic health records had an explicit sepsis code [4].

Under the tentative assumption that the coding practices in our single center study would be representative for all German hospitals causing an underestimation using explicit sepsis coding and an overestimation using implicit sepsis coding, the average number of severe sepsis cases for Germany for the period between 2007 and 2013 would range between 277-372/100.000 persons. This would still be considerably less compared to the 687/100 000 traditional severe sepsis and 780/100 000 “sepsis-3” definition cases for Sweden. A US-wide projection of a recent national report using electronic health record data of representative US hospitals resulted in 1.67 million cases which corresponds to an incidence of approx. 517/100 000 persons [4]. Using EHR data, incidence rates of severe sepsis remained stable over time between 2009 and 2014, as did the mortality defined as combined outcome of in-hospital death (15% in 2014) and discharge to hospice (6.2% in 2014). Comparing temporary dynamics of hospital discharge diagnoses in the same patient cohort, they found an increase in coded severe sepsis incidence by an annual mean of 10% and a decrease in mortality by a mean of 7% annually. Given these differences, the coding of less severe sepsis cases may be a main contributor to the trends observed using hospital discharge data and EHR-based clinical data may provide more objective estimates for sepsis surveillance.

Similarly, we observed a significant increase of age- and sex-standardized sepsis incidence in Germany irrespective of the coding strategy, which went along with a moderate decline in mortality. However, there are also important differences compared to the findings in Sweden [17] and the US [4]. First, incidence rates for severe sepsis are substantially lower in Germany. This may be due to regulations for the coding of sepsis to avoid overcoding: Patients without blood culture sampling cannot be coded as sepsis. In case of negative blood cultures, patients need to fulfill 4/4 SIRS criteria. Since in only about 2/3 of cases blood cultures are collected and only half deliver positive results [25], this may result in an underestimation of sepsis cases. Above that, 12% of severe sepsis patients have negative SIRS criteria [26] and thus are excluded from coding by the German coding regulations. In Germany, the overall incidence of hospital-treated infection, and the proportion of coded sepsis among patients with infectious diseases is substantially lower than in Sweden and the US: Nearly 30% of infection patients who received intravenous antibiotics in Sweden were retrospectively diagnosed as sepsis patients with organ dysfunction according to the new sepsis definitions (780 of 2425/100,000 population) [17]. In the US, this ratio was 15% (535 of 3480/100,000 population) [27] compared to 7% in Germany [28]. These differences may result from a considerable undercoding of sepsis cases and contribute to the lower incidence rates observed in our study. As our analyses have shown, the number of severe sepsis cases increased when sepsis was identified by the combination of infection and organ dysfunction (implicit strategy), which is less prone to coding bias. This confirms the recent results of a US-study showing that trends in sepsis over time match those of infection cases requiring mechanical ventilation [27]. In contrast other studies using administrative data [4, 29], mortality rates were higher in Germany and only declined moderately over time. These differences may arise from several factors, including the higher age of our patients (median 72 years compared to a mean of 66 years in the US [4]), longer hospital length of stay, potentially reflecting varying discharge policies between countries, and that only a minimal, stable proportion of patients was discharged to hospice (0.2% of severe sepsis patients [28] compared to 6.2% in the US [4]). The admission rates to ICU facilities were decreasing over time in severe sepsis patients. Furthermore, differences in health care, population structure and the lack of nationwide quality improvement initiatives for sepsis in Germany are possible factors that may contribute to the between-country differences in sepsis mortality over time.

With its broad, representative sample, this validation study has an unique approach and provides a comprehensive assessment of the accuracy of ICD case identification for sepsis. No preliminary selection of cases according to the presence or absence of relevant ICD codes was applied, minimizing the risk of selection bias [30] compared to previous validation studies, which reported heterogeneous validity of sepsis case identification strategies in administrative data [13]. Our results are consistent with a previous validation study by Iwashyna et al., yielding better positive predictive values for explicit, and higher sensitivity for implicit coding strategies [14]. Profound variability in sepsis incidence and mortality depending on the ICD abstraction strategy was observed in different studies [12, 31]. For implicit coding, higher incidence due to false positives and lower mortality rates result from a missing causal relation between infection and organ dysfunction. Explicit coding strategies identify fewer cases with higher mortality, because sepsis is not always coded properly especially when less severe [32, 33], and relevant organ dysfunctions are not coded as found in our analyses.

It should be clear that a perfect agreement between cases identified from administrative data and from clinical criteria cannot be expected [34]. When using a framework proposed by Angus et al. [34], it is obvious that both approaches have strengths and weaknesses in different domains. Their usefulness depends highly on the intended purpose, with an important role for administrative data based strategies especially in quality improvement [35].

Despite our comprehensive approach, our study has several limitations. Since the DRG-statistics is a nation-wide all-payer inpatient database, the accuracy of coding is likely to vary between hospitals, thus validation results are not be fully generalizable and a transfer of conclusions is only possible to a limited degree. Given specific coding regulations in Germany, results may not be applicable to other countries. Our validation was based on retrospective manual patient chart review rather than on prospective assessment and is therefore limited by the quality of documentation. Clinical diagnosis according to the current sepsis definitions including SIRS criteria as gold standard may miss 8–10% of septic patients as found in Australia [26].

Further multicenter evaluation is needed to obtain comprehensive data on coding quality, allowing better estimates of the national burden of sepsis and the development of a reliable surveillance coding strategy that agrees with clinical case identification.

## Supporting information

S1 File Supplementary methodological information. (DOCX)

S2 File Supplementary results. (DOCX)

## References

[1] Fleischmann C, Scherag A, Adhikari NK, Hartog CS, Tsaganos T, Schlattmann P, et al Assessment of Global Incidence and Mortality of Hospital-treated Sepsis. Current Estimates and Limitations. American journal of respiratory and critical care medicine. 2016;193(3):259–72. Epub 2015/09/29. 10.1164/rccm.201504-0781OC . 26414292

[2] World Health Organisation Executive Board (EB140/12). Improving the prevention, diagnosis and clinical management of sepsis. 2017 [2017/06/20]. http://apps.who.int/gb/ebwha/pdf_files/EB140/B140_12-en.pdf.

[3] Reinhart K, Daniels R, Kissoon N, Machado FR, Schachter RD, Finfer S. Recognizing Sepsis as a Global Health Priority—A WHO Resolution. The New England journal of medicine. 2017 Epub 2017/06/29. 10.1056/NEJMp1707170 . 28658587

[4] Rhee C, Dantes R, Epstein L, Murphy DJ, Seymour CW, Iwashyna TJ, et al Estimating The National Burden Of Sepsis Using Clinical Data. American journal of respiratory and critical care medicine. 2017;195:A5010.

[5] Angus DC, Linde-Zwirble WT, Lidicker J, Clermont G, Carcillo J, Pinsky MR. Epidemiology of severe sepsis in the United States: analysis of incidence, outcome, and associated costs of care. Critical care medicine. 2001;29(7):1303–10. Epub 2001/07/11. . 11445675 10.1097/00003246-200107000-00002

[6] Krumholz HM, Wang Y, Mattera JA, Wang YF, Han LF, Ingber MJ, et al An administrative claims model suitable for profiling hospital performance based on 30-day mortality rates among patients with an acute myocardial infarction. Circulation. 2006;113(13):1683–92. 10.1161/CIRCULATIONAHA.105.611186 16549637

[7] Krumholz HM, Wang Y, Mattera JA, Wang YF, Han LF, Ingber MJ, et al An administrative claims model suitable for profiling hospital performance based on 30-day mortality rates among patients with heart failure. Circulation. 2006;113(13):1693–701. 10.1161/CIRCULATIONAHA.105.611194 16549636

[8] Bratzler DW, Normand SLT, Wang Y, O’Donnell WJ, Metersky M, Han LF, et al An Administrative Claims Model for Profiling Hospital 30-Day Mortality Rates for Pneumonia Patients. Plos One. 2011;6(4). 10.1371/journal.pone.0017401 21532758 PMC3075250

[9] Qualitätsmedizin I. G-IQI: German Inpatient Quality Indicators. Version 4.2—Band 1: Definitionshandbuch für das Datenjahr 2015 2015 [2015-11-01]. https://opus4.kobv.de/opus4-tuberlin/frontdoor/index/index/docId/7007.

[10] ICOSMOS-Studie. http://www.icosmos.uniklinikum-jena.de.

[11] Martin GS, Mannino DM, Eaton S, Moss M. The epidemiology of sepsis in the United States from 1979 through 2000. The New England journal of medicine. 2003;348(16):1546–54. Epub 2003/04/18. 10.1056/NEJMoa022139 . 12700374

[12] Lagu T, Rothberg MB, Shieh MS, Pekow PS, Steingrub JS, Lindenauer PK. What is the best method for estimating the burden of severe sepsis in the United States? Journal of critical care. 2012;27(4):414 e1–9. Epub 2012/04/21. 10.1016/j.jcrc.2012.02.004 . 22516143

[13] Jolley RJ, Sawka KJ, Yergens DW, Quan H, Jette N, Doig CJ. Validity of administrative data in recording sepsis: a systematic review. Crit Care. 2015;19:139 Epub 2015/04/19. 10.1186/s13054-015-0847-3 . 25887596 PMC4403835

[14] Iwashyna TJ, Odden A, Rohde J, Bonham C, Kuhn L, Malani P, et al Identifying patients with severe sepsis using administrative claims: patient-level validation of the angus implementation of the international consensus conference definition of severe sepsis. Medical care. 2014;52(6):e39–43. Epub 2012/09/25. 10.1097/MLR.0b013e318268ac86 . 23001437 PMC3568444

[15] Singer M, Deutschman CS, Seymour CW, Shankar-Hari M, Annane D, Bauer M, et al The Third International Consensus Definitions for Sepsis and Septic Shock (Sepsis-3). JAMA: the journal of the American Medical Association. 2016;315(8):801–10. Epub 2016/02/24. 10.1001/jama.2016.0287 . 26903338 PMC4968574

[16] Henriksen DP, Laursen CB, Jensen TG, Hallas J, Pedersen C, Lassen AT. Incidence rate of community-acquired sepsis among hospitalized acute medical patients-a population-based survey*. Critical care medicine. 2015;43(1):13–21. Epub 2014/09/25. 10.1097/CCM.0000000000000611 . 25251760

[17] Mellhammar L, Wullt S, Lindberg A, Lanbeck P, Christensson B, Linder A. Sepsis Incidence: A Population-Based Study. Open forum infectious diseases. 2016;3(4):ofw207. Epub 2016/12/13. 10.1093/ofid/ofw207 . 27942538 PMC5144652

[18] Bone RC, Balk RA, Cerra FB, Dellinger RP, Fein AM, Knaus WA, et al Definitions for sepsis and organ failure and guidelines for the use of innovative therapies in sepsis. The ACCP/SCCM Consensus Conference Committee. American College of Chest Physicians/Society of Critical Care Medicine. Chest. 1992;101(6):1644–55. Epub 1992/06/01. . 1303622 10.1378/chest.101.6.1644

[19] Reinhart K, Brunkhorst FM, Bone HG, Bardutzky J, Dempfle CE, Forst H, et al Prevention, diagnosis, therapy and follow-up care of sepsis: 1st revision of S-2k guidelines of the German Sepsis Society (Deutsche Sepsis-Gesellschaft e.V. (DSG)) and the German Interdisciplinary Association of Intensive Care and Emergency Medicine (Deutsche Interdisziplinare Vereinigung fur Intensiv- und Notfallmedizin (DIVI)). German medical science: GMS e-journal. 2010;8:Doc14 Epub 2010/07/16. 10.3205/000103 . 20628653 PMC2899863

[20] Quan H, Sundararajan V, Halfon P, Fong A, Burnand B, Luthi JC, et al Coding algorithms for defining comorbidities in ICD-9-CM and ICD-10 administrative data. Medical care. 2005;43(11):1130–9. Epub 2005/10/15. . 16224307 10.1097/01.mlr.0000182534.19832.83

[21] Falotico R, Quatto P. On avoiding paradoxes in assessing inter-rater agreement. Statistica Applicata—Italian Journal of Applied Statistics. 2010;22(2):151–60.

[22] Fleiss JL. Measuring Nominal Scale Agreement among Many Raters. Psychol Bull. 1971;76(5):378–82. 10.1037/H0031619

[23] Lex A, Gehlenborg N, Strobelt H, Vuillemot R, Pfister H. UpSet: Visualization of Intersecting Sets. IEEE transactions on visualization and computer graphics. 2014;20(12):1983–92. 10.1109/TVCG.2014.2346248 . 26356912 PMC4720993

[24] Team RDC. R: A Language and Environment for Statistical Computing. Vienna, Austria: R Foundation for Statistical Computing; 2014.

[25] Bloos F, Thomas-Ruddel D, Ruddel H, Engel C, Schwarzkopf D, Marshall JC, et al Impact of compliance with infection management guidelines on outcome in patients with severe sepsis: a prospective observational multi-center study. Crit Care. 2014;18(2):R42 Epub 2014/03/05. 10.1186/cc13755 . 24589043 PMC4057316

[26] Kaukonen KM, Bailey M, Pilcher D, Cooper DJ, Bellomo R. Systemic inflammatory response syndrome criteria in defining severe sepsis. The New England journal of medicine. 2015;372(17):1629–38. Epub 2015/03/18. 10.1056/NEJMoa1415236 . 25776936

[27] Walkey AJ, Lagu T, Lindenauer PK. Trends in sepsis and infection sources in the United States. A population-based study. Annals of the American Thoracic Society. 2015;12(2):216–20. Epub 2015/01/09. 10.1513/AnnalsATS.201411-498BC . 25569845 PMC4342831

[28] Mikolajetz A, Fleischmann C, Schwarzkopf D, Thomas-Rüddel D, Reinhart K. Trends in infection and sepsis incidence and mortality in Germany (abstract). 38th International Symposium on Intensive Care and Emergency Medicine (ISICEM). 2018;Brussels, March 20–23 2018.

[29] Lagu T, Rothberg MB, Shieh MS, Pekow PS, Steingrub JS, Lindenauer PK. Hospitalizations, costs, and outcomes of severe sepsis in the United States 2003 to 2007. Critical care medicine. 2012;40(3):754–61. Epub 2011/10/04. 10.1097/CCM.0b013e318232db65 . 21963582

[30] Weiner MG, Garvin JH, Ten Have TR. Assessing the accuracy of diagnostic codes in administrative databases: the impact of the sampling frame on sensitivity and specificity. AMIA Annual Symposium proceedings / AMIA Symposium AMIA Symposium. 2006:1140. Epub 2007/01/24. 17238759. PMC1839455 17238759

[31] Liu V, Escobar GJ, Greene JD, Soule J, Whippy A, Angus DC, et al Hospital Deaths in Patients With Sepsis From 2 Independent Cohorts. JAMA: the journal of the American Medical Association. 2014;312(1):90–2. Epub 2014/05/20. 10.1001/jama.2014.5804 . 24838355

[32] Ollendorf DA, Fendrick AM, Massey K, Williams GR, Oster G. Is sepsis accurately coded on hospital bills? Value in health: the journal of the International Society for Pharmacoeconomics and Outcomes Research. 2002;5(2):79–81. Epub 2002/03/29. 10.1046/j.1524-4733.2002.52013.x . 11918823

[33] Whittaker SA, Mikkelsen ME, Gaieski DF, Koshy S, Kean C, Fuchs BD. Severe sepsis cohorts derived from claims-based strategies appear to be biased toward a more severely ill patient population. Critical care medicine. 2013;41(4):945–53. Epub 2013/02/07. 10.1097/CCM.0b013e31827466f1 . 23385099 PMC3810475

[34] Angus DC, Seymour CW, Coopersmith CM, Deutschman CS, Klompas M, Levy MM, et al A Framework for the Development and Interpretation of Different Sepsis Definitions and Clinical Criteria. Critical care medicine. 2016;44(3):e113–21. 10.1097/CCM.0000000000001730 . 26901559 PMC4765912

[35] Seymour CW, Coopersmith CM, Deutschman CS, Gesten F, Klompas M, Levy M, et al Application of a Framework to Assess the Usefulness of Alternative Sepsis Criteria. Critical care medicine. 2016;44(3):e122–30. Epub 2016/02/24. 10.1097/CCM.0000000000001724 . 26901560 PMC4765919

