# Supplementary material for: Comparing the validity of different ICD coding abstraction strategies for sepsis case identification in German claims data
Source: PLoS One. 2018 Jul 30;13(7):e0198847. doi: 10.1371/journal.pone.0198847 (PMC6066203; doi:10.1371/journal.pone.0198847)
Supplement: S1 File — (DOCX) [file pone.0198847.s001.docx]

**S1 File: Supplementary Methodological Information**

This supplement forms part of the original submission:

**Sepsis in administrative data: Benchmarking and validation of ICD coding strategies**

Fleischmann-Struzek Carolin*, Thomas-Rüddel Daniel O*, Schettler Anna, Schwarzkopf Daniel, Stacke Angelika, Seymour Christopher W, Haas Christoph, Dennler Ulf, Reinhart Konrad.

* contributed equally

**1.1. Database description: DRG statistics**

Since 2004, reimbursement for hospital treatment is mandatorily billed by DRG (diagnosis-related groups) by private and statutory health insurances comprising 78.9 million insurance holders in Germany. Complete data from all discharges is submitted annually to the Federal Statistical Office (FSO) and is accessible in the DRG statistics about 18 months later.

Access to the database is granted via remote data processing. Thereby the user sends analysis scripts to the FSO and receives back anonymous summary statistics or other results. By this process there is no need for further data protection considerations.

There are some exceptions, but the DRG statistics include nearly all acute hospital cases in Germany. Under certain circumstances separate hospital treatments for the same disease in the same hospital in a short timeframe are combined to a single billing case and documented as such in the statistics.

Not included in the statistics are military personnel treated in military hospitals, but it includes civilians treated in military hospitals and military personnel treated in civilian hospitals. Also not included are work-related accidents and recognized occupational diseases treated in hospitals of the Hospital Group of the Statutory Accident Insurance (BG Hospitals), but all such cases treated in other hospitals and all other cases treated in those hospitals. Also not included are inmates treated in prison hospitals. Cases from psychiatric and psychosomatic facilities are includes but not accessible for analysis [[1](#_ENREF_1)]. During the study years 2007-2013, it contained data on 14.6 to 16.1 million adult hospital cases from 1725 to 1550 hospitals annually. Each inpatient treatment is coded with one principal ICD-10-GM (International Statistical Classification of Diseases and Related Health Problems, 10. Revision, German Modification) diagnosis, up to 89 ICD-10-GM secondary diagnoses, hospital mortality and length of stay, patient demographics and up to 100 procedures coded by OPS codes (Classification of Operations and Procedures, modified codes of the International Classification of Procedures in Medicine, ICPM). Age, sex, hospital mortality, length of stay and mechanical ventilation are also included. Diagnoses and procedures are not time stamped in any way.

**1.2. Selection of relevant ICD codes for sepsis case identification**

22 international studies applying ICD abstraction strategies for sepsis case identification in administrative data on a population level [[2-23](#_ENREF_2)] were identified in a systematic literature search as part of a systematic review [[24](#_ENREF_24)]. Those were analyzed for the infection, sepsis and organ dysfunction codes used. A comprehensive list of codes was extracted and expert reviewed. Codes for three main coding strategies were selected and translated from ICD-9 to ICD-10-GM (German modification).

**1.3. Validation**

Validation of ICD coding strategies was performed based on the billing data and patient files for patients admitted to a large university hospital in Germany (Jena University Hospital). All adult hospitalizations admitted between 2007 and 2013 to general medical, surgical, neurology, urology and gynecology service were stratified according to ICU treatment and a hospital length of stay (LOS) of up to or more than six days.

Chart review was conducted by three trained investigators (AS, ASt, CF), which were blinded to the patients’ administrative billing data. Every chart was reviewed for the presence of infection and sepsis independently by two investigators using a structured protocol, discrepancies were resolved by discussion and a third review by an intensive care fellow with extensive experience in chart review (DTR). Sepsis was defined according to modified ACCP/SCCM consensus criteria [[25](#_ENREF_25),[26](#_ENREF_26)] as SIRS due to microbiologically proven or clinically suspected infection. Organ dysfunctions were evaluated for diagnosis of severe sepsis and were considered if most likely caused by sepsis. For each patient, underlying infection, blood cultures, need of ICU treatment/mechanical ventilation/renal replacement therapy/other organ replacement, SOFA/APACHE II/SAPSII scores and several laboratory parameters were extracted. Following the analysis, false negatives and false positives were reviewed to determine reasons for misclassification. To evaluate the impact of the new sepsis-3 definitions [[27-29](#_ENREF_27)], all infection cases underwent a second review according to the definitions proposed by the taskforce (change in SOFA >=2 points in the 24h after infection compared to the 48h prior to the onset of infection, lactate values for septic shock). If data were missing, no SOFA points were assigned.

**1.3. Clinical sepsis codes (ICD-10, GM)**

R65.0! - SIRS due to infection without organ dysfunction, R65.1! - SIRS due to infection with organ dysfunction, R57.2 - septic shock

**1.4. Microbiological and clinical sepsis codes, explicit approach) (ICD-10-GM)**

A02.1 - Salmonella sepsis, A20.0 - Bubonic plague, A20.7 - Septicemic plague, A21.7 - Generalized tularemia, A22.7 - Anthrax sepsis, A24.1 - Acute and fulminating melioidosis, A26.7 - Erysipelothrix sepsis, A28.2 - Extraintestinal yersiniosis, A32.7 - Listerial sepsis, A39.2 - Acute meningococcemia, A39.3 - Chronic meningococcemia, A39.4 - Meningococcemia, unspecified, A39.1 - Waterhouse-Friderichsen syndrome, A40.- - Streptococcal sepsis, A41.-- Other sepsis, A42.7 - Actinomycotic sepsis, A48.3 - Toxic shock syndrome, B00.7 - Herpetic septicemia, A54.8 - Other gonococcal infections (sepsis), B37.7 - Candidal sepsis, B37.6 - Candidal endocarditis, B49 - Unspecified mycosis (Fungemia), A49.9 - Bacterial infection, unspecified (bacteremia), R65.0! - Systemic inflammatory response syndrome (SIRS) due to infection without organ dysfunction, R65.1! - Systemic inflammatory response syndrome (SIRS) due to infection with organ dysfunction, R57.2 - Septic shock

**1.5. Organ dysfunction codes (ICD-10-GM)**

Cardiovascular: I95.9 - Hypotension, unspecified, R57.8 - Other shock, R57.9 - Shock, unspecified,

Respiratory: J96.- -Acute respiratory failure with hypoxia, not elsewhere classified, J96.9 - Respiratory failure, unspecified with hypoxia, J80.- - Acute respiratory distress syndrome, J98.4 - Other disorders of lung, R06.0 - Dyspnea, R06.8 - Other abnormalities of breathing

Central nervous system: F05 - Delirium (not alcohol- or drug-induced), G93.1 - Anoxic brain damage, not elsewhere classified, G93.4 - Encephalopathy, unspecified, R40.- - Somnolence, stupor and coma

Renal: N17.- - Acute kidney failure, N19.- Unspecified kidney failure

Metabolic: E87.2 - Acidosis

Hematologic: D65.- - Disseminated intravascular coagulation [defibrination syndrome], D68.8 - Other specified coagulation defects, D68.9 - Coagulation defect, unspecified, D69.5- - Secondary thrombocytopenia, D69.6- - Thrombocytopenia, unspecified

Hepatic: K72.0 - Acute and subacute hepatic failure, K76.2 - Central hemorrhagic necrosis of liver, K72.7-! - Hepatic encephalopathy and hepatic coma, K76.3 - Infarction of liver

Other: R65.1! - SIRS due to infection with organ dysfunction, R57.2 - septic shock

**1.6. Infection codes, implicit approach (ICD-10-GM)**

A00.- - Cholera, A01.- - Typhoid and paratyphoid fevers, A02.- - Other salmonella infections, A03.- - Shigellosis, A04.- - Other bacterial intestinal infections, A05.- Other bacterial foodborne intoxications, not elsewhere classified, A06.- - Amebiasis, A07.- - Other protozoal intestinal diseases, A08.- - Viral and other specified intestinal infections, A09.- - Infectious gastroenteritis and colitis, unspecified, A20.- - Plague, A21.- - Tularemia, A22.- - Anthrax, A23.- - Brucellosis, A24.- - Glanders and melioidosis, A25.- Rat-bite fevers, A26.- - Erysipeloid, A28.- - Other zoonotic bacterial diseases, not elsewhere classified, A32.- - Listeriosis, A36.- - Diphtheria, A37.- - Whooping cough, A38 - Scarlet fever, A46 – Erysipelas, A39.- - Meningococcal infection, A40.- - Streptococcal sepsis, A41.- - Other sepsis, A42.- -Actinomycosis, A43.- - Nocardiosis, A44.- - Bartonellosis, A48.- - Other bacterial diseases, not elsewhere classified, A49.- - Bacterial infection of unspecified site, A54.- - Gonococcal infection, A27.- - Leptospirosis, A69.0 - Necrotizing ulcerative stomatitis, A69.1 - Other Vincent's infections, A69.8 - Other specified spirochetal infections, A69.9 - Spirochetal infection, unspecified, A69.2 - Lyme disease, B35.- - Dermatophytosis, B36.- - Other superficial mycoses, B37.- - Candidiasis, B38.- - Coccidioidomycosis, B39.- - Histoplasmosis, B40.- - Blastomycosis, B49 - Unspecified mycosis, B41.- - Paracoccidioidomycosis, B42.- - Sporotrichosis, B43.- - Chromomycosis and pheomycotic abscess, B44.- - Aspergillosis, B45.- - Cryptococcosis, B46.- - Zygomycosis, B47.- - Mycetoma, B48.- - Other mycoses, not elsewhere classified, G00.- - Bacterial meningitis, not elsewhere classified, G01* - Meningitis in bacterial diseases classified elsewhere, G02.-* - Meningitis in other infectious and parasitic diseases classified elsewhere, G03.- - Meningitis due to other and unspecified causes, G04.- - Encephalitis, myelitis and encephalomyelitis, G05.-* - Encephalitis, myelitis and encephalomyelitis in diseases classified elsewhere, G06.- - Intracranial and intraspinal abscess and granuloma, G07* - Intracranial and intraspinal abscess and granuloma in diseases classified elsewhere, G08 - Intracranial and intraspinal phlebitis and thrombophlebitis, I30.- - Acute pericarditis, I32.-* - Pericarditis in diseases classified elsewhere, I33.- - Acute and subacute endocarditis, I39.-* - Endocarditis and heart valve disorders in diseases classified elsewhere, I40.- - Acute myocarditis, I41.-* - Myocarditis in diseases classified elsewhere, I80.- - Phlebitis and thrombophlebitis, J00 - Acute nasopharyngitis [common cold], J01.- - Acute sinusitis, J02.- - Acute pharyngitis, J03.- - Acute tonsillitis, J04.- - Acute laryngitis and tracheitis, J06.- - Acute upper respiratory infections of multiple and unspecified sites, J05.- - Acute obstructive laryngitis [croup] and epiglottitis, J09 - Influenza due to certain identified influenza viruses, J10.- - Influenza due to other identified influenza virus, J11.- - Influenza due to unidentified influenza virus, J12.-- Viral pneumonia, not elsewhere classified, J13 - Pneumonia due to Streptococcus pneumoniae, J14 - Pneumonia due to Hemophilus influenzae, J15.- - Bacterial pneumonia, not elsewhere classified, J16.- - Pneumonia due to other infectious organisms, not elsewhere classified, J17.-* - Pneumonia in diseases classified elsewhere, J18.- - Pneumonia, unspecified organism, J20.- - Acute bronchitis, J21.- - Acute bronchiolitis, J22 - Unspecified acute lower respiratory infection, J44.0- - Chronic obstructive pulmonary disease with acute lower respiratory infection, J44.1- - Chronic obstructive pulmonary disease with acute exacerbation,unspecified, J47 - Bronchiectasis, J86.- - Pyothorax, J85.- Abscess of lung and mediastinum, K35.- - Acute appendicitis, K37 - Unspecified appendicitis, K36 - Other appendicitis, K57.12 - Diverticulitis of small intestine without perforation or abscess without bleeding, K57.02 - Diverticulitis of small intestine with perforation and abscess, without bleeding, K57.13 - Diverticulitis of small intestine without perforation or abscess with bleeding, K57.03 - Diverticulitis of small intestine with perforation, abscess and bleeding, K57.22 - Diverticulitis of large intestine with perforation and abscess without bleeding, K57.32 - Diverticulitis of large intestine without perforation or abscess without bleeding, K57.23 - Diverticulitis of large intestine with perforation, abscess and bleeding, K57.33 - Diverticulitis of large intestine without perforation or abscess with bleeding, K57.42 - Diverticulitis of both small and large intestine with perforation and abscess, without bleeding, K57.43 - Diverticulitis of both small and large intestine without perforation or abscess without bleeding, K57.52 - Diverticulitis of both small and large intestine with perforation, abscess and bleeding, K57.53 - Diverticulitis of both small and large intestine without perforation or abscess with bleeding, K57.82 - Diverticulitis of intestine, part unspecified, with perforation and abscess without bleeding, K57.83 - Diverticulitis of intestine, part unspecified, with perforation, abscess and bleeding, K57.92 - Diverticulitis of intestine, part unspecified, without perforation or abscess without bleeding, K57.93 - Diverticulitis of intestine, part unspecified, without perforation or abscess with bleeding, K61.- - Abscess of anal and rectal regions, K65.- - Peritonitis, K67.-* -Disorders of peritoneum in infectious diseases classified elsewhere, K63.0 - Abscess of intestine, K63.1 - Perforation of intestine (nontraumatic), K75.0 - Abscess of liver, K75.1 -Phlebitis of portal vein, K81.0 - Acute cholecystitis, N10 - Acute tubulo-interstitial nephritis, N11.- - Chronic tubulo-interstitial nephritis, N12 - Tubulo-interstitial nephritis, not specified as acute or chronic, N15.1- - Renal and perinephric abscess, N15.9 - Renal tubulo-interstitial disease, unspecified, N16.-* - Renal tubulo-interstitial disorders in infectious and parasitic diseases classified elsewhere, N28.8 - Other specified disorders of kidney and ureter, N34.- - Urethritis and urethral syndrome, N30.- - Cystitis, N39.0 - Urinary tract infection, site not specified, N41.- - Inflammatory diseases of prostate, N45.- - Orchitis and epididymitis, N51.-* - Disorders of male genital organs in diseases classified elsewhere, N48.2 - Other inflammatory disorders of penis, N49.- - Inflammatory disorders of male genital organs, not elsewhere classified, N70.- - Salpingitis and oophoritis, N71.- - Inflammatory disease of uterus, except cervix, N72 - Inflammatory disease of cervix uteri, N73.- - Other female pelvic inflammatory diseases, N74.-* - Female pelvic inflammatory disorders in diseases classified elsewhere, N75.- - Diseases of Bartholin's glands, N76.- - Other inflammation of vagina and vulva, N77.-* - Vulvovaginal ulceration and inflammation in diseases classified elsewhere, N61 - Inflammatory disorders of breast, L03.- - Phlegmon, L04.- - Acute lymphadenitis, L08.- - Other local infections of skin and subcutaneous tissue, L88 - Pyoderma gangrenosum, L05.- - Pilonidal cyst and sinus, M00.- - Pyogenic arthritis, M01.-* - Direct infections of joint in infectious and parasitic diseases classified elsewhere, M86.- - Osteomyelitis, A49.9 - Bacteremia, unspecified, T82.6 - Infection and inflammatory reaction due to cardiac valve prosthesis, T82.7 - Infection and inflammatory reaction due to other cardiac and vascular devices, implants and grafts, T83.5 - Infection and inflammatory reaction due to prosthetic device, implant and graft in urinary system, T83.6 - Infection and inflammatory reaction due to prosthetic device, implant and graft in genital tract, T84.5 - Infection and inflammatory reaction due to unspecified internal joint prosthesis, T84.6 - Infection and inflammatory reaction due to internal fixation device of unspecified site, T84.7 - Infection and inflammatory reaction due to other internal orthopedic prosthetic devices, implants and grafts, T85.7- - Infection and inflammatory reaction due to other internal prosthetic devices, implants and grafts, T81.4 - Infection following a procedure, T80.2 - Infection following infusion, transfusion or therapeutic injection, T88.0 - Infection following immunization, R65.0! - Systemic inflammatory response syndrome (SIRS) due to infection without organdysfunction, R65.1! - Systemic inflammatory response syndrome (SIRS) due to infection with organdysfunction, R57.2 - Septic shock, O75.3 - Other infection during labor, O85 - Puerperal sepsis, O03.- - Spontaneous abortion, O04.- - Medically induced abortion, O05.- - Other abortion, O06.- - Abortion, unspecified, O07.- - Failed attempted termination of pregnancy, O08.0 - Genital tract and pelvic infection following abortion or ectopic or molar pregnancy, A15.- - Respiratory tuberculosis, confirmed , A16.- - Respiratory tuberculosis, not confirmed, A17.- - Tuberculosis of nervous system, A18.- - Tuberculosis of other organs, A19.- - Miliary tuberculosis, B00. - Herpesviral infection, B50.- - Plasmodium falciparum malaria, B51.- - Plasmodium vivax malaria, B52.- - Plasmodium malariae malaria, B53.- - Other specified malaria, B54 - Unspecified malaria, A90 - Dengue fever [classic dengue], A91 - Dengue hemorrhagic fever

**1.7. OPS Codes (The Operationen- und Prozedurenschlüssel Codes, German modification of the International Classification of Procedures in Medicine)**

ICU treatment: OPS 8-890

Mechanical ventilation: OPS 8-71

Renal replacement therapy: OPS 8-854, 8-854, 8-855, 8-856, 8-857**1.8. Structured validation protocol**

**1) INFECTION**

| a | Microbiologically proven: ⊕ blood culture, wound secretion, smear, sputum, tracheal secretion, BAL, urine, faeces, liquor, others: | Yes | No | n/a |
| --- | --- | --- | --- | --- |
|  |  | Causative microorganism(s):  ____________________________ ____________________________ | | |
| b | Clinically suspected: by imaging (Sono, CT, MRT, others), urine dipstick test, surgical confirmation, others | Yes | No | n/a |
| c | Documented administration of antibiotics | Yes | No | n/a |
|  | **= Infection?** | **Yes** | **No** | **n/a** |

| a | respiratory | Yes | No | n/a |
| --- | --- | --- | --- | --- |
| b | abdominal | Yes | No | n/a |
| c | genitourinary | Yes | No | n/a |
| d | skin/soft tissue | Yes | No | n/a |
| e | pregnancy-related | Yes | No | n/a |
| f | device-related | Yes | No | n/a |
| g | unknown/other |  | | |

**2) SIRS-CRITERIA**

| a | Fever (≥38°C) or hypothermia (≤36°C)  Temperature: | Yes | No | n/a |
| --- | --- | --- | --- | --- |
| b | Tachycardia (Heart rate ≥90 /min)  Heart rate: | Yes | No | n/a |
| c | Tachypnoa (frequency ≥20/min) or hyperventilation (PaCO2 ≤4.3 kPa/ ≤33 mmHg)  frequency:  PaCO2 breathing ambient air: | Yes | No | n/a  n/a  n/a |
| d | Leukocytosis (≥12000/mm3) or Leukopenia (≤4000/mm3) or ≥10% immature neutrophils in differential blood count  Leukocytes:  Neutrophils: | Yes | No | n/a  n/a  n/a |
|  | **Number of positive SIRS criteria** | **n=** |  |  |

**3) ORGAN DYSFUNCTION**

| a | Acute encephalopathy (reduced alertness, disorientation, agitation, delirium) | Yes | No | n/a |
| --- | --- | --- | --- | --- |
| b | Relative or absolute thrombocytopenia (decrease in platelet count by more than 30% in 24 h or platelet count of ≤100.000/mm^3^, without acute hemorrhage or immunological causes)  Platelet count: | Yes | No | n/a  n/a |
| c | Arterial hypoxemia (PaO2 ≤10 kPa (≤75 mmHg) while breathing ambient air or PaO2/FiO2-ratio ≤33 kPa (≤250 mmHg) on oxygen administration, without clinically manifest heart- or lung disease)  PaO2 on oxygen administration:  FiO2 on oxygen administration | Yes | No | n/a  n/a |
| d | Renal Impairment (Diuresis ≤0.5 ml/kg/h for at least 2 h despite adequate volume resuscitation or increase in serum creatinine level > 2x the upper limit of normal (UNL)  Creatinine:  Diuresis: | Yes | No | n/a  n/a  n/a |
| e | Metabolic acidosis (Base Excess ≤-5 mmol/l or Lactate concentration > 1,5× ULN)  Base excess:  Lactate concentration: | Yes | No | n/a |
|  | **= Organ dysfunction?** | **Yes** | **No** | **n/a** |
|  | Art. hypotension (> 1 h systolic arterial BP ≤90 mmHg or MAP ≤65 mmHg despite adequate volume resuscitation or vasopressor administration to maintan target systolic BP of ≥90 mmHg or MAP ≥65mmHg)  BP systolic:  BP diastolic: | Yes | No | n/a |
|  | **= Shock?** | **Yes** | **No** | **n/a** |

**4) DIAGNOSIS**

| **a** | **Sepsis** | **Yes** | **No** | n/a |
| --- | --- | --- | --- | --- |
| **b** | **Severe Sepsis** | **Yes** | **No** | n/a |
| **c** | **Septic Shock** | **Yes** | **No** | n/a |

**In case of sepsis/severe sepsis/septic shock:**

**5) BLOOD CULTURES**

| a | Blood cultures were taken | Yes | No | n/a |
| --- | --- | --- | --- | --- |
| b | Blood cultures had a positive result  Causative microorganism(s): | Yes | No | n/a |

**6) INTENSIVE CARE TREATMENT**

| a | Patient received intensive care treatment | Yes | No | n/a |
| --- | --- | --- | --- | --- |
| b | ICU length of stay |  | days | n/a |
| c | Patient required mechanical ventilation | Yes | No | n/a |
| d | Patient required dialysis | Yes | No | n/a |
| e | Patient required other organ dysplacement | Yes | No | n/a |

In case of severe sepsis/septic shock:

**7) SOFA-SCORE/SAPS II**

|  | **Time:** |  | Value | Unit |
| --- | --- | --- | --- | --- |
| a | Bilirubin | - |  |  |
| b | Vasopressors |  |  |  |
| c | GCS | - |  |  |
| d | Sodium |  |  |  |
| e | Potassium |  |  |  |
| f | Bicarbonate |  |  |  |
| g | Urea |  |  |  |
| h | Chronic diseases (Metastatic cancer, hematologic malignancy, AIDS) |  | | |
| i | Type of admission (scheduled surgical, medical, unscheduled surgical) |  | | |

**During ICU stay:
SOFA at admission: SOFA_maximum:
SAPSII_at admission: SAPSII_maximum:
APACHE_at admission: APACHE_maximum:**

**Supplemental References:**

1. Hochgürtel T, Lösch T (2012) Analysen mit der DRG-Statistik Entwicklung und Anwendung eines Hilfsinstruments zur effizienten Erstellung neuer Variablen in der DRG-Statistik. Wiesbaden: Statistische Ämter des Bundes und der Länder.

2. Angus DC, Linde-Zwirble WT, Lidicker J, Clermont G, Carcillo J, et al. (2001) Epidemiology of severe sepsis in the United States: analysis of incidence, outcome, and associated costs of care. Crit Care Med 29: 1303-1310.

3. Martin GS, Mannino DM, Eaton S, Moss M (2003) The epidemiology of sepsis in the United States from 1979 through 2000. N Engl J Med 348: 1546-1554.

4. Danai PA, Sinha S, Moss M, Haber MJ, Martin GS (2007) Seasonal variation in the epidemiology of sepsis. Crit Care Med 35: 410-415.

5. Iwashyna TJ, Odden A, Rohde J, Bonham C, Kuhn L, et al. (2014) Identifying patients with severe sepsis using administrative claims: patient-level validation of the angus implementation of the international consensus conference definition of severe sepsis. Med Care 52: e39-43.

6. Shen HN, Lu CL, Yang HH (2010) Epidemiologic trend of severe sepsis in Taiwan from 1997 through 2006. Chest 138: 298-304.

7. Dombrovskiy VY, Martin AA, Sunderram J, Paz HL (2007) Rapid increase in hospitalization and mortality rates for severe sepsis in the United States: a trend analysis from 1993 to 2003. Crit Care Med 35: 1244-1250.

8. Lagu T, Rothberg MB, Shieh MS, Pekow PS, Steingrub JS, et al. (2012) Hospitalizations, costs, and outcomes of severe sepsis in the United States 2003 to 2007. Crit Care Med 40: 754-761.

9. Lagu T, Rothberg MB, Shieh MS, Pekow PS, Steingrub JS, et al. (2012) What is the best method for estimating the burden of severe sepsis in the United States? J Crit Care 27: 414 e411-419.

10. Gaieski DF, Edwards JM, Kallan MJ, Carr BG (2013) Benchmarking the incidence and mortality of severe sepsis in the United States. Crit Care Med 41: 1167-1174.

11. Seymour CW, Iwashyna TJ, Cooke CR, Hough CL, Martin GS (2010) Marital status and the epidemiology and outcomes of sepsis. Chest 137: 1289-1296.

12. Andreu Ballester JC, Ballester F, Gonzalez Sanchez A, Almela Quilis A, Colomer Rubio E, et al. (2008) Epidemiology of sepsis in the Valencian Community (Spain), 1995-2004. Infect Control Hosp Epidemiol 29: 630-634.

13. Hall MJ, Williams SN, DeFrances CJ, Golosinskiy A (2011) Inpatient care for septicemia or sepsis: a challenge for patients and hospitals. NCHS Data Brief: 1-8.

14. (1990) Increase in National Hospital Discharge Survey rates for septicemia--United States, 1979-1987. MMWR Morb Mortal Wkly Rep 39: 31-34.

15. Kumar G, Kumar N, Taneja A, Kaleekal T, Tarima S, et al. (2011) Nationwide trends of severe sepsis in the 21st century (2000-2007). Chest 140: 1223-1231.

16. Wilhelms SB, Huss FR, Granath G, Sjoberg F (2010) Assessment of incidence of severe sepsis in Sweden using different ways of abstracting International Classification of Diseases codes: difficulties with methods and interpretation of results. Crit Care Med 38: 1442-1449.

17. Barnato AE, Alexander SL, Linde-Zwirble WT, Angus DC (2008) Racial variation in the incidence, care, and outcomes of severe sepsis: analysis of population, patient, and hospital characteristics. Am J Respir Crit Care Med 177: 279-284.

18. Liu V, Escobar GJ, Greene JD, Soule J, Whippy A, et al. (2014) Hospital Deaths in Patients With Sepsis From 2 Independent Cohorts. JAMA 312(1):90-2.

19. Flaatten H (2004) Epidemiology of sepsis in Norway in 1999. Crit Care 8: R180-184.

20. Sundararajan V, Macisaac CM, Presneill JJ, Cade JF, Visvanathan K (2005) Epidemiology of sepsis in Victoria, Australia. Crit Care Med 33: 71-80.

21. Heublein S, Hartmann M, Hagel S, Hutagalung R, Brunkhorst F (2013) Epidemiology of sepsis in German hospitals derived from administrative databases. Infection 41: S71.

22. Henriksen DP, Laursen CB, Jensen TG, Hallas J, Pedersen C, et al. (2015) Incidence rate of community-acquired sepsis among hospitalized acute medical patients-a population-based survey. Crit Care Med 43: 13-21.

23. Wang HE, Shapiro NI, Angus DC, Yealy DM (2007) National estimates of severe sepsis in United States emergency departments. Crit Care Med 35: 1928-1936.

24. Fleischmann C, Scherag A, Adhikari NKJ, Hartog CS, Tsaganos T, et al. (2016) Assessment of Global Incidence and Mortality of Hospital-treated Sepsis. American Journal of Respiratory and Critical Care Medicine 193: 259-272.

25. Bone RC, Balk RA, Cerra FB, Dellinger RP, Fein AM, et al. (1992) Definitions for sepsis and organ failure and guidelines for the use of innovative therapies in sepsis. The ACCP/SCCM Consensus Conference Committee. American College of Chest Physicians/Society of Critical Care Medicine. Chest 101: 1644-1655.

26. Levy MM, Fink MP, Marshall JC, Abraham E, Angus D, et al. (2003) 2001 SCCM/ESICM/ACCP/ATS/SIS International Sepsis Definitions Conference. Crit Care Med 31: 1250-1256.

27. Seymour CW, Liu VX, Iwashyna TJ, Brunkhorst FM, Rea TD, et al. (2016) Assessment of Clinical Criteria for Sepsis: For the Third International Consensus Definitions for Sepsis and Septic Shock (Sepsis-3). JAMA 315: 762-774.

28. Shankar-Hari M, Phillips GS, Levy ML, Seymour CW, Liu VX, et al. (2016) Developing a New Definition and Assessing New Clinical Criteria for Septic Shock: For the Third International Consensus Definitions for Sepsis and Septic Shock (Sepsis-3). JAMA 315: 775-787.

29. Singer M, Deutschman CS, Seymour CW, Shankar-Hari M, Annane D, et al. (2016) The Third International Consensus Definitions for Sepsis and Septic Shock (Sepsis-3). JAMA 315: 801-810.
