# Supplementary material for: Comparing the validity of different ICD coding abstraction strategies for sepsis case identification in German claims data
Source: PLoS One. 2018 Jul 30;13(7):e0198847. doi: 10.1371/journal.pone.0198847 (PMC6066203; doi:10.1371/journal.pone.0198847)
Supplement: S2 File — (DOCX) [file pone.0198847.s002.docx]

**S2 File: Supplementary results**

**Results from a second review of incorrectly coded sepsis cases – Identification of reasons for incorrect coding**

A) Implicit coding strategy

False-positive cases identified by implicit sepsis coding (infection and organ dysfunction) resulted from concurrence of infection and organ dysfunction without causality (51%) or that infection or organ dysfunctions were not identifiable retrospectively from manual patient chart review (40%). 9% were SIRS-negative and were not classified as severe sepsis according to our clinical criteria. In false-negative patients, organ dysfunction, infection or both were not coded in 67%, 14% and 17%, respectively. Encephalopathy, respiratory failure and hypotension were coded in 10.5%, 56.1% and 36.7% of cases, respectively.

B) Explicit coding strategy

In cases identified by the explicit coding strategy that were coded false positive (n=13), organ dysfunction was not caused by infection (n=3), infection or organ dysfunction were not identifiable by manual patient chart review (n=7) or patients were SIRS negative and thus not classified as sepsis (n=3). **Supplementary tables**

Table A: Hospital cases ≥18y in Germany per year by ICD code abstraction strategies

| Characteristic | **ICD abstraction strategies for sepsis** | | **ICD abstraction strategies for severe sepsis** | | |
| --- | --- | --- | --- | --- | --- |
|  | R codes (R65.0!, R65.1!, R57.2) | explicit sepsis coding | R codes (R65.1!, R57.2) | explicit sepsis coding +odf | implicit sepsis coding (inf+odf) |
| 2007 | 105.797 | 181.440 | 52.213 | 98.587 | 474.094 |
| 2008 | 113.810 | 184.849 | 60.811 | 106.216 | 520.856 |
| 2009 | 134.792 | 197.827 | 70.006 | 119.221 | 605.201 |
| 2010 | 156.435 | 214.533 | 86.223 | 136.444 | 688.562 |
| 2011 | 176.111 | 225.335 | 94.661 | 145.746 | 749.146 |
| 2012 | 194.736 | 242.535 | 103.327 | 159.136 | 818.361 |
| 2013 | 216.541 | 264.991 | 113.537 | 176.607 | 929.291 |

Table B: Hospital deaths ≥18y in Germany per year by ICD code abstraction strategies

| Characteristic | **ICD abstraction strategies for sepsis** | | **ICD abstraction strategies for severe sepsis** | | |
| --- | --- | --- | --- | --- | --- |
|  | R codes (R65.0!, R65.1!, R57.2) | explicit sepsis coding | R codes (R65.1!, R57.2) | explicit sepsis coding +odf | implicit sepsis coding (inf+odf) |
| 2007 | 35.799 | 53.504 | 26.380 | 42.530 | 105.820 |
| 2008 | 37.937 | 54.202 | 30.454 | 45.022 | 114.544 |
| 2009 | 43.561 | 56.436 | 34.018 | 48.387 | 127.332 |
| 2010 | 50.294 | 60.441 | 41.766 | 53.790 | 139.040 |
| 2011 | 53.204 | 60.628 | 44.156 | 54.975 | 143.476 |
| 2012 | 56.153 | 62.898 | 46.373 | 57.451 | 151.617 |
| 2013 | 60.790 | 67.310 | 50.036 | 62.097 | 167.687 |

Table C: Hospital mortality rate of patients ≥18y in Germany per year by ICD code abstraction strategies

| Characteristic | **ICD abstraction strategies for sepsis** | | **ICD abstraction strategies for severe sepsis** | | |
| --- | --- | --- | --- | --- | --- |
|  | R codes (R65.0!, R65.1!, R57.2) | explicit sepsis coding | R codes (R65.1!, R57.2) | explicit sepsis coding +odf | implicit sepsis coding (inf+odf) |
| 2007 | 34% | 29% | 51% | 43% | 22% |
| 2008 | 33% | 29% | 50% | 42% | 22% |
| 2009 | 32% | 29% | 49% | 41% | 21% |
| 2010 | 32% | 28% | 48% | 39% | 20% |
| 2011 | 30% | 27% | 47% | 38% | 19% |
| 2012 | 29% | 26% | 45% | 36% | 19% |
| 2013 | 28% | 25% | 44% | 35% | 18% |

Table D: Hospital incidence ≥18y in Germany per 100 000 persons ≥18y per year by ICD code abstraction strategies

| Characteristic | **ICD abstraction strategies for sepsis** | | **ICD abstraction strategies for severe sepsis** | | |
| --- | --- | --- | --- | --- | --- |
|  | R codes (R65.0!, R65.1!, R57.2) | explicit sepsis coding | R codes (R65.1!, R57.2) | explicit sepsis coding +odf | implicit sepsis coding (inf+odf) |
| 2007 | 155 | 266 | 77 | 144 | 695 |
| 2008 | 167 | 271 | 89 | 155 | 762 |
| 2009 | 197 | 290 | 102 | 175 | 886 |
| 2010 | 229 | 314 | 126 | 199 | 1007 |
| 2011 | 262 | 335 | 141 | 217 | 1115 |
| 2012 | 289 | 360 | 153 | 236 | 1214 |
| 2013 | 320 | 391 | 168 | 261 | 1372 |

Table E: Hospital deaths ≥18y in Germany per 100 000 persons ≥18y per year of discharge by different ICD code abstraction strategies

| Characteristic | **ICD abstraction strategies for sepsis** | | **ICD abstraction strategies for severe sepsis** | | |
| --- | --- | --- | --- | --- | --- |
|  | R codes (R65.0!, R65.1!, R57.2) | explicit sepsis coding | R codes (R65.1!, R57.2) | explicit sepsis coding +odf | implicit sepsis coding (inf+odf) |
| 2007 | 52 | 78 | 39 | 62 | 155 |
| 2008 | 56 | 79 | 45 | 66 | 168 |
| 2009 | 64 | 83 | 50 | 71 | 186 |
| 2010 | 74 | 88 | 61 | 79 | 203 |
| 2011 | 79 | 90 | 66 | 82 | 214 |
| 2012 | 83 | 93 | 69 | 85 | 225 |
| 2013 | 90 | 99 | 74 | 92 | 248 |

Table F: Hospital incidence ≥18y in Germany per 100 000 persons ≥18y per year by ICD code abstraction strategies, adjusted to the German population ≥18y in 2010

| Characteristic | **ICD abstraction strategies for sepsis** | | **ICD abstraction strategies for severe sepsis** | | |
| --- | --- | --- | --- | --- | --- |
|  | R codes (R65.0!, R65.1!, R57.2) | explicit sepsis coding | R codes (R65.1!, R57.2) | explicit sepsis coding +odf | implicit sepsis coding (inf+odf) |
| 2007 | 162 | 279 | 80 | 151 | 733 |
| 2008 | 172 | 280 | 92 | 161 | 792 |
| 2009 | 200 | 294 | 104 | 177 | 902 |
| 2010 | 229 | 314 | 126 | 199 | 1007 |
| 2011 | 259 | 331 | 139 | 214 | 1099 |
| 2012 | 282 | 351 | 149 | 230 | 1181 |
| 2013 | 309 | 379 | 162 | 252 | 1322 |

Table G: Hospital deaths ≥18y in Germany per 100 000 persons ≥18y per year of discharge by different ICD code abstraction strategies, adjusted to the German population ≥18y in 2010

| Characteristic | **ICD abstraction strategies for sepsis** | | **ICD abstraction strategies for severe sepsis** | | |
| --- | --- | --- | --- | --- | --- |
|  | R codes (R65.0!, R65.1!, R57.2) | explicit sepsis coding | R codes (R65.1!, R57.2) | explicit sepsis coding +odf | implicit sepsis coding (inf+odf) |
| 2007 | 56 | 83 | 41 | 66 | 166 |
| 2008 | 58 | 83 | 46 | 69 | 176 |
| 2009 | 65 | 84 | 51 | 72 | 191 |
| 2010 | 74 | 88 | 61 | 79 | 203 |
| 2011 | 78 | 89 | 65 | 81 | 210 |
| 2012 | 81 | 91 | 67 | 83 | 218 |
| 2013 | 86 | 95 | 71 | 88 | 237 |

Table H: Comparison of clinical sepsis diagnosis according to ACCP/SCCM consensus criteria [1, 2] vs. “sepsis-3” [3] criteria by retrospective chart review

|  |  | Sepsis 3 Definitions | | |
| --- | --- | --- | --- | --- |
|  | Characteristic | Infection (w/o ODF) | Sepsis | Septic Shock |
| Consensus Definitions | Infection (w/o SIRS) | 132 | 3 | 0 |
|  | Sepsis | 6 | 64 | 0 |
|  | Severe Sepsis | 0 | 17 | 0 |
|  | Septic Shock | 0 | 5 | 59 |

**Supplementary figures**

Figure A

Hospital deaths ≥18y in Germany per 100 000 persons ≥18y per year of discharge by different ICD code abstraction strategies, adjusted to the German population ≥18y in 2010

Figure B


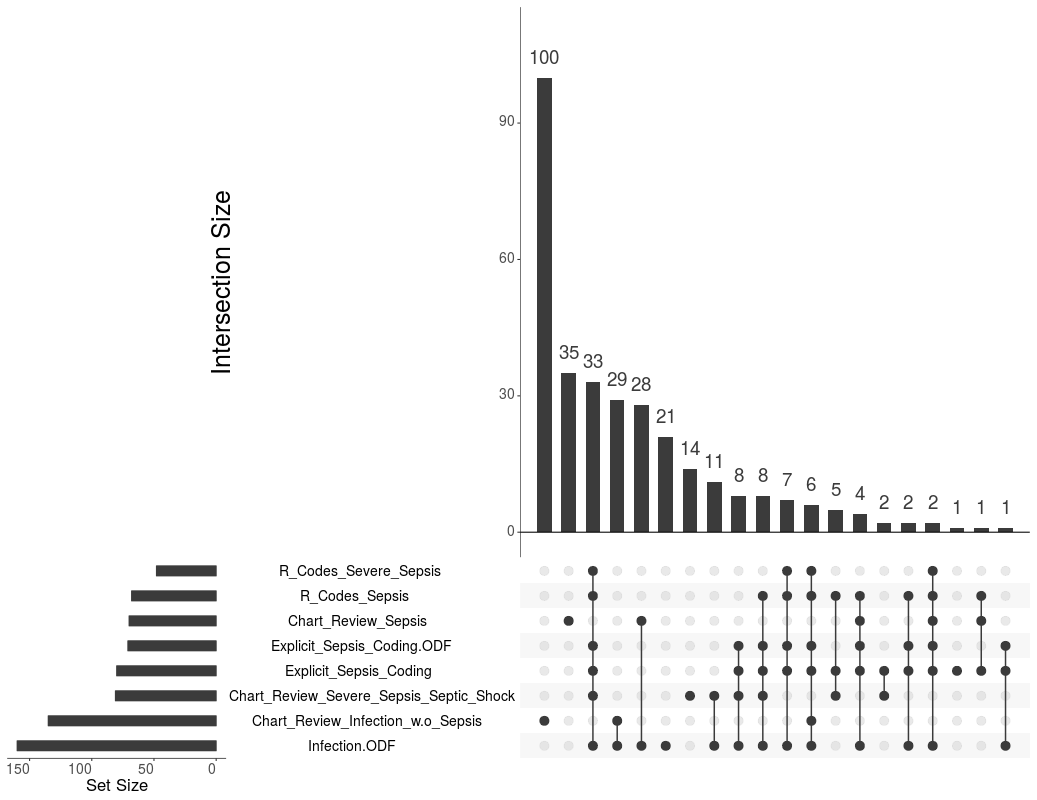


Intersecting sets of all reviewed cases with at least a single positive result from ICD code abstraction strategies or at least a diagnosis of infection in chart review; **horizontal bars represent the numbers of patients identified by a specific coding strategy or diagnosed by chart review to have infection with or without (severe) sepsis; vertical bars represent the number of patients diagnosed by only one or several coding strategies and/or chart review indicated by dots with connecting lines;
e.g. 29 patients had infection without sepsis on chart review but coding for infection&ODF but no R-codes or explicit sepsis codes (generated with UpSetR[4])**

**References**

1. Bone RC, Balk RA, Cerra FB, Dellinger RP, Fein AM, Knaus WA, et al. Definitions for sepsis and organ failure and guidelines for the use of innovative therapies in sepsis. The ACCP/SCCM Consensus Conference Committee. American College of Chest Physicians/Society of Critical Care Medicine. Chest. 1992;101(6):1644-55. Epub 1992/06/01. PubMed PMID: 1303622.

2. Levy MM, Fink MP, Marshall JC, Abraham E, Angus D, Cook D, et al. 2001 SCCM/ESICM/ACCP/ATS/SIS International Sepsis Definitions Conference. Crit Care Med. 2003;31(4):1250-6. Epub 2003/04/12. doi: 10.1097/01.CCM.0000050454.01978.3B. PubMed PMID: 12682500.

3. Singer M, Deutschman CS, Seymour CW, Shankar-Hari M, Annane D, Bauer M, et al. The Third International Consensus Definitions for Sepsis and Septic Shock (Sepsis-3). JAMA : the journal of the American Medical Association. 2016;315(8):801-10. Epub 2016/02/24. doi: 10.1001/jama.2016.0287. PubMed PMID: 26903338.

4. Lex A, Gehlenborg N, Strobelt H, Vuillemot R, Pfister H. UpSet: Visualization of Intersecting Sets. IEEE transactions on visualization and computer graphics. 2014;20(12):1983-92. doi: 10.1109/TVCG.2014.2346248. PubMed PMID: 26356912; PubMed Central PMCID: PMC4720993.
